# RIP140 and LCoR expression in gastrointestinal cancers

**DOI:** 10.18632/oncotarget.22686

**Published:** 2017-11-25

**Authors:** Mouna Triki, Dorra Ben Ayed-Guerfali, Ines Saguem, Slim Charfi, Lobna Ayedi, Tahia Sellami-Boudawara, Vincent Cavailles, Raja Mokdad-Gargouri

**Affiliations:** ^1^ IRCM (Institute of Cancer Research of Montpellier), INSERM U1194, Montpellier University, Montpellier, France; ^2^ Center of Biotechnology of Sfax, Laboratory of Eukaryotic Molecular Biotechnology, Sfax University, Sfax, Tunisia; ^3^ Department of Anatomopathology, Habib Bourguiba Hospital, Sfax, Tunisia

**Keywords:** gastrointestinal cancer, RIP140, LCoR, immunohistochemistry, patient survival

## Abstract

The transcription coregulators RIP140 and LCoR are part of a same complex which controls the activity of various transcription factors and cancer cell proliferation. In this study, we have investigated the expression of these two genes in human colorectal and gastric cancers by immunohistochemistry. In both types of tumors, the levels of RIP140 and LCoR appeared highly correlated. Their expression tended to decrease in colorectal cancer as compared to adjacent normal tissues but was found higher in gastric cancer as compared to normal stomach. RIP140 and LCoR expression correlated with TNM and tumor differentiation. Significant correlations were observed with expression levels of key proteins involved in tumor progression and invasion namely E-cadherin and Cyclooxygenase-2. Survival analysis showed that patients with LCoR^low^/RIP140^high^ colorectal tumors have a significant prolonged overall and disease-free survival. In gastric cancer, high LCoR expression was identified as an independent marker of poor prognosis suggesting a key role in this malignancy. Altogether, these results demonstrate that RIP140 and LCoR have a prognostic relevance in gastrointestinal cancers and could represent new potential biomarkers in these tumors.

## INTRODUCTION

Gastrointestinal cancers (GICs) correspond to a diverse set of diseases of the gastrointestinal tract which include colorectal cancer (CRC) and gastric cancer (GC) and are among the most common human cancers [1]. Colorectal carcinogenesis relies on step-wise genetic events leading from early adenomatous lesions to invasive carcinomas and metastatic cancers [2]. Although chromosomal and microsatellite instability pathways constitute the major genetic alteration events in CRC [3][4], epigenetic alterations play a key role in the colorectal carcinogenesis [5]. The prognosis for CRC has been improved over the past decade, especially by treatment advances, but only about 30% of patients are diagnosed at early stages, and the prognosis still poor for patients with advanced stage of the disease [3]. GCs account for over 70% of all cancers in developing countries [1] and are subdivided into two morphologically distinct groups corresponding to diffuse and intestinal cancers according to Lauren’s classification [6]. The pathogenesis of GC is closely related to environmental factors particularly *Helicobacter pylori* infection [7], and genetic predisposition occurred in a subset of GC cases [8]. Because of the prognosis variability within a clinical or pathological stage of GC, it is significant to identify specific biological markers for a better management of patients with more aggressive behavior [9].

More recently, genome wide analyses have further characterized GIC heterogeneity by defining different molecular subtypes through common expression signatures [10]. More precisely, this allowed the identification of subgroups with expression of mesenchymal genes, extensive immune infiltration or metabolic dysregulation. In addition to these subtypes, both CRC and GC exhibit specific molecular subtypes with characteristic features linked to the tissue of origin. Such approaches might lead to the identification of novel molecular prognostic markers which could improve our understanding of the molecular mechanisms underlying GIC tumorigenesis.

Altered gene expression is a hallmark of cancer and the identification of factors which account for the dysregulation of transcriptional programs represent a key step in the understanding of cancer pathogenesis [11]. Transcription coactivators and corepressors are involved in the fine tuning of transcription factor activity and clearly participate in establishing new patterns of gene expression in cancer cells [12]. Amongst others, RIP140 (Receptor Interacting Protein of 140kDa) also called NRIP1 (Nuclear Receptor-Interacting Protein 1) and LCoR (Ligand-dependent CoRepressor) act mainly as transcriptional repressors of nuclear receptors and other transcription factors (for a review see [13]). These two transcription coregulators act by recruiting histone deacetylases and C-terminal binding proteins [14][15][16]. RIP140 and LCoR were both isolated in interaction with agonist- activated ERα [15][17]. However, in addition to ligand-activated nuclear receptors, they also interact with other transcription factors. For instance, LCoR interacts with Kruppel-like factor 6 (KLF6) [18] and KRAB-associated protein 1 (KAP1) [19], whereas RIP140 has been reported to bind and regulate E2Fs [20], NFKB [21] or β-catenin [22].

Several studies reported that the two transcription coregulators might play important roles in human cancers. RIP140 is required for mammary gland development [23] and regulates breast cancer cell proliferation and tumor progression [24]. Its strong impact on intestinal homeostasis and tumorigenesis has been unraveled using molecular and cellular approaches, transgenic mouse models and human CRC biopsies [25]. Interestingly, we recently reported that RIP140 directly interacted with LCoR and was necessary for LCoR inhibition of gene expression and cell proliferation [26]. Moreover, RIP140 and LCoR expression were strongly correlated in breast cancer cell lines and biopsies and correlated with overall survival of patients with breast cancer thus highlighting their strong interplay for the control of gene expression and cell proliferation in breast cancer cells. Finally, a very recent study confirmed the relevance of LCoR in breast cancer by demonstrating that it inhibits mammary cancer stem cells activity [27].

In this study, we investigated by immunohistochemistry (IHC) the expression of RIP140 and LCoR in CRC and GC. We showed that the levels of the two transcription factors were highly correlated in both tumors. Interestingly, whereas their expression tended to decrease in CRC as compared to adjacent normal tissues, an increase of RIP140 and LCoR expression was noticed in GC as compared to normal stomach. RIP140 and LCoR expression were correlated with various clinicopathological parameters in CRC and/or GC including TNM stage and tumor differentiation. Moreover, in GICs, the expression of RIP140 and LCoR correlated with E-cadherin or COX-2 (Cyclooxygenase-2). Univariate and multivariate survival analyses indicated that high LCoR expression was an independent marker of poor prognosis in GC suggesting a key role in this malignancy.

## RESULTS

### Immunodetection of RIP140 and LCoR in gastrointestinal cancers

**Figure 1:**
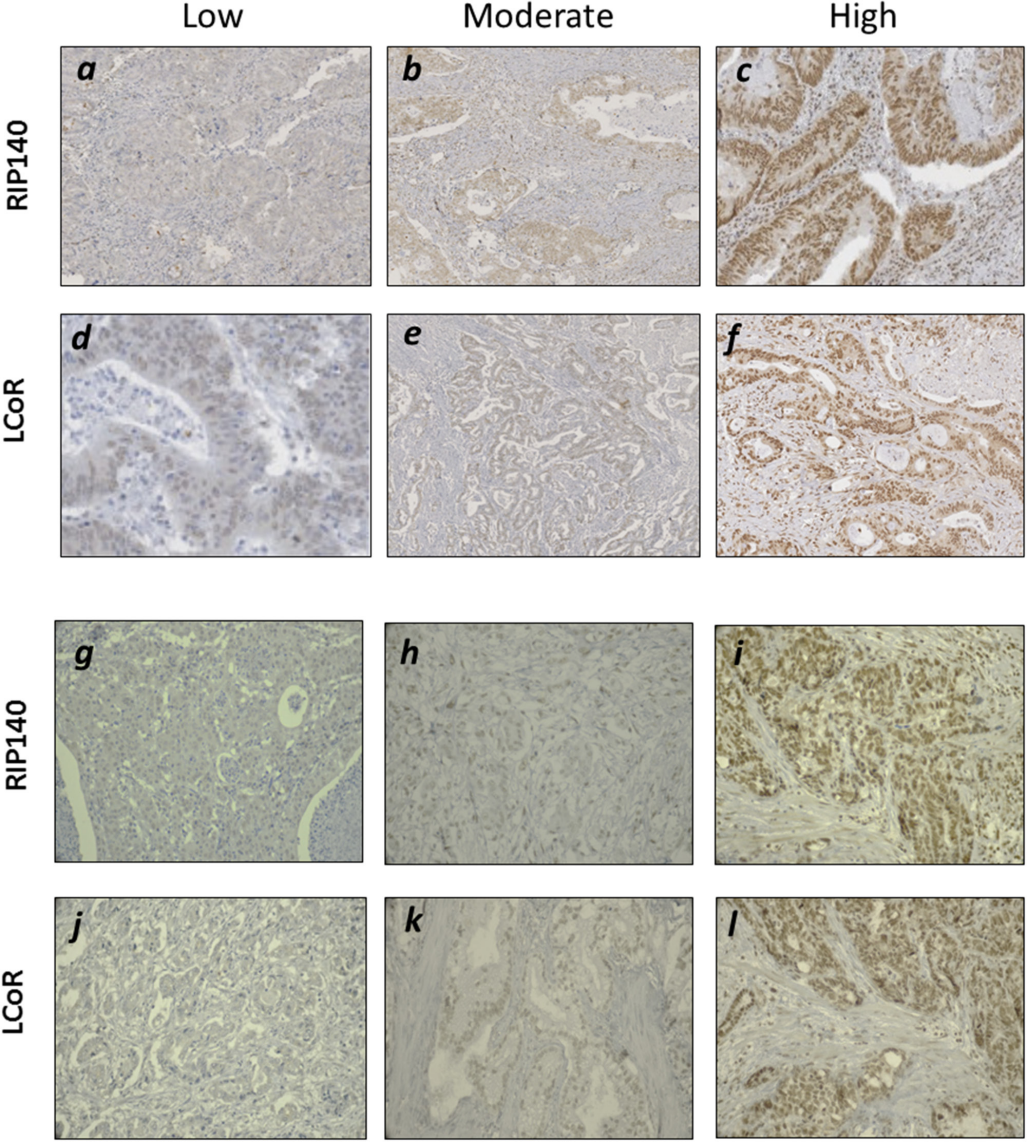
Immunohistochemical staining of RIP140 and LCoR in GICs Representative images for low, moderate and high nuclear IHC staining of RIP140 **(a-c)** and LCoR **(d-f)** in CRC specimen. Same in GC for low, moderate and high nuclear staining of RIP140 **(g-i)** and LCoR **(j-l)**.

The expression of RIP140 and LCoR was evaluated by IHC in 102 and 41 specimens of CRC and GC, respectively. Representative examples of RIP140 and LCoR immunostaining in are shown in Figure 1. Based on the immunostaining scores (IS), quantification of RIP140 and LCoR expression in CRC showed that RIP140 expression levels were high in 56.9%, moderate in 29.4% and low (negative or weak) in 13.7% of tumor tissues (Table 2A and Figure 1a-1f). Amongst the 102 CRC cases, only 99 gave a convincing LCoR IHC staining. Low LCoR expression was observed in most cases (54.5%) whereas 29.3%, and 16.2% of tumor tissues displayed intense or moderate LCoR immunostaining, respectively (Table 2A and Figure 1a-1f). In GC, the expression of RIP140 was intense in 80.5% of tumors while only 18 cases (43.9%) exhibited strong LCoR immunostaining (Table 2A and Figure 1g-1l). When examining the expression of both transcription factors, tumors negative for both RIP140 and LCoR were not found frequent in CRC (1.8%) or GC (2.5%) (Table 2B). Most of the tumors expressed both RIP140 and LCoR with 12.4% and 19.5% of CRC and GC respectively harboring the maximal IS. Noticeably, a significant proportion of CRC (21%) and GC (10%) only exhibited RIP140 immunostaining (Table 2B).

**Table 1:** Clinicopathological characteristics of CRC and GC patients

*Variable*	*CRC (n=102)*	*GC (n=41)*
***Age***		
***≤60***	***39***	***19***
***>60***	***63***	***22***
***Sex***	***47***	
***Male***	***55***	***22***
***Differentiation***	***61***	
***Poor***	***37***	***21***
***TNM stage***		
***I-II***	***41***	***2***
***III***	***42***	***10***
***IV***	***17***	***16***
***Tumor site***		
***Colon***	***64***	***-***
***Rectum***	***37***	***-***
***Anatomical site***		
***Antrum***	***-***	***23***
***Body***	***-***	***12***
***Cardia***	***-***	***4***
***Tumor size***		
***≤5cm***	***53***	***3***
***>5cm***	***48***	***31***
***Lymphovascular***		
***invasion***		
***Yes***	***32***	***9***
***No***	***67***	***32***
***Histological type***		
***Intestinal***	***-***	***23***
***Diffuse***	***-***	***18***
***H. pylori***		
***Positive***	***-***	***10***
***Negative***	***-***	***13***

**Table 2:** Expression of RIP140 and LCoR in GICs

A							
		*RIP140*			*LCoR*		
		*Low*	*Moderate*	*High*	*Low*	*Moderate*	*High*
***CRC***	***n***	***14***	***30***	***58***	***55***	***17***	***30***
	***%***	***13.7***	***29.4***	***56.9***	***54.5***	***16.2***	***29.3***
***GC***	***n***	***4***	***4***	***33***	***15***	***8***	***18***
	***%***	***9.8***	***9.8***	***80.5***	***36.6***	***19.5***	***43.9***

B							
	*CRC*				*GC*			
*RIP140*	*-*	*+*	*+*	*Max*	*-*	*+*	*+*	*Max*
*LCoR*	*-*	*-*	*+*	*Max*	*-*	*-*	*+*	*Max*
***n***	***3***	***34***	***104***	***20***	***1***	***4***	***27***	***9***
***%***	***1.8***	***21***	***64.5***	***12.4***	***2.5***	***10***	***67.5***	***19.5***

**Figure 2:**
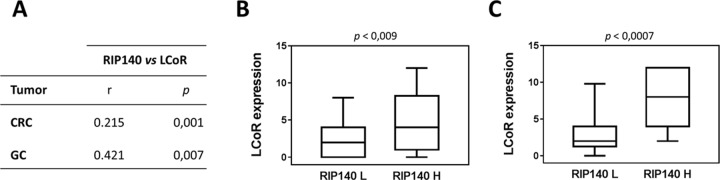
Association between RIP140 and LCoR expression in colorectal and gastric cancers **(A)** The correlations between RIP140 and LCoR IS have been analyzed in CRC and GC as described in Material and methods using SPSS. The correlation coefficient and *p* values are indicated. **(B)** Boxplot representation showing the significant association between LCoR expression in groups with low (L) and high (H) RIP140 expression in CRCs (*p* = 0.009). **(C)** Same representation in GC biopsies (*p* = 0.0007).

**Figure 3:**
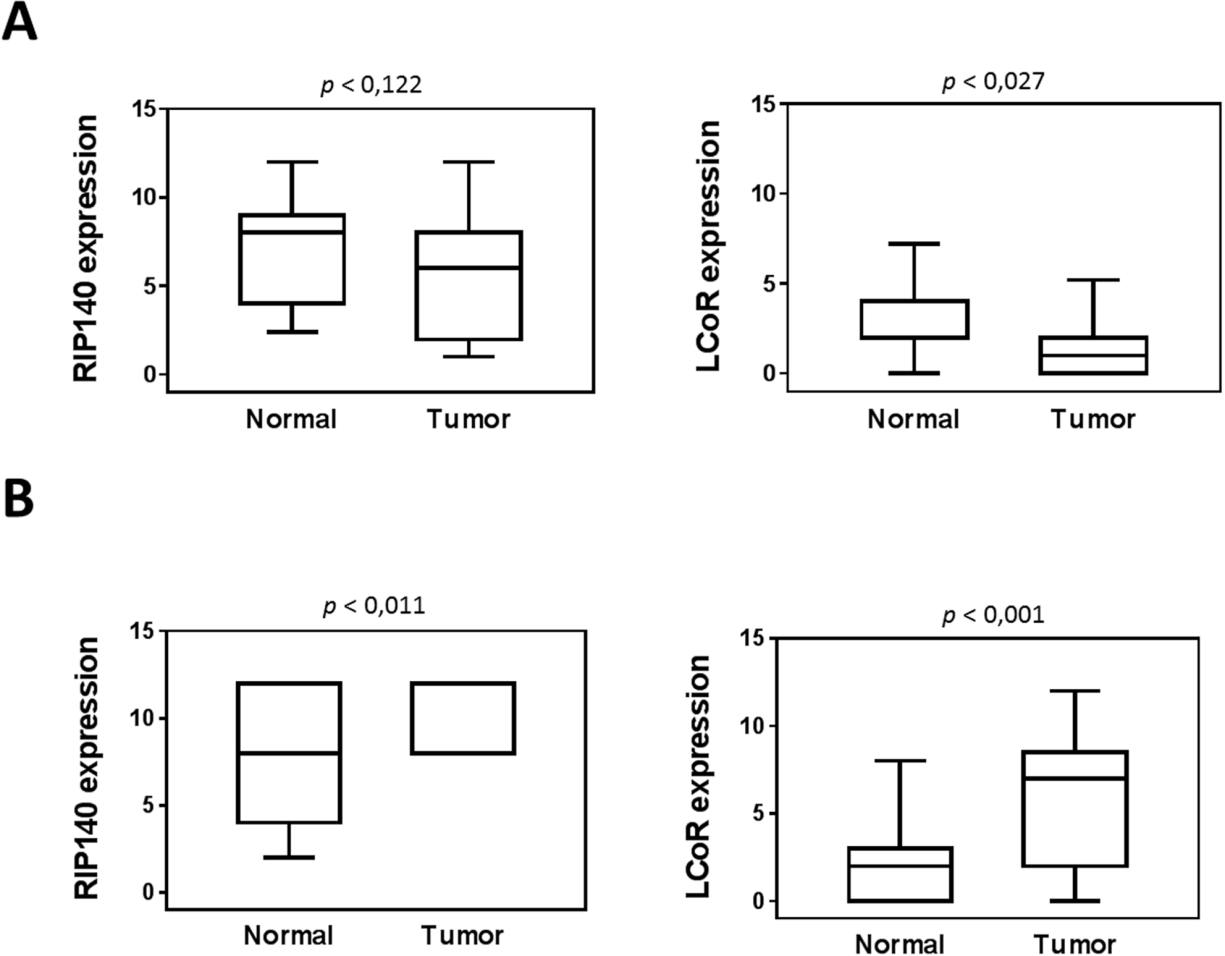
Expression of RIP140 and LCoR in tumoral and adjacent normal mucosa RIP140 (left panel) and LCoR (right panel) protein expression were quantified in twenty-three tumoral tissues and adjacent normal mucosa from colorectal **(A)** and gastric **(B)** biopsies. All correlations were performed using the nonparametric Mann–Whitney *U* test. Boxplots show the median and interquartile range (box) and the 10-90 percentiles (whiskers).

Very interestingly, a strong positive association was observed between RIP140 and LCoR protein levels in both CRC and GC (Figure 2A-2C). Moreover, when we compared the expression of the two proteins in tumors with the adjacent normal tissues, we only observed a significant decrease for LCoR (Figure 3A). On the contrary, RIP140 and LCoR levels increase in GC as compared to adjacent normal tissues (p<0.011 p<0.001 respectively) (Figure 3B and Supplementary Figure 1).

### Association of RIP140 and LCoR with other proteins and with clinical parameters

We next asked whether the expression levels of RIP140 and/or LCoR were correlated with the expression of other proteins previously quantified in the same cohorts of CRC and GC patients [28][29][30][31][32]. As shown in Table 3, in CRC and GC, RIP140 correlated positively with E-cadherin (p=0.049 and p=0.013, respectively) and COX-2 (p=0.021 and p=0.004, respectively). LCoR also associated with E-cadherin in GC (p=0.043) but was inversely correlated in CRC (p=0.006). Finally, LCoR also correlated with p53 only in CRC (p=0.003) (Table 3).

**Table 3:** Correlation between RIP140 and LCoR with other proteins in CRC and GC

*Proteins*	*CRC*		*GC*	
	*RIP140*	*LCoR*	*RIP140*	*LCoR*
***β-catenin***	***p=0.104***	***p=0.43***	***p=0.204***	***p=0.749***
	***r=0.078***	***r=0.07***	***r=0.06***	***r=-0.101***
***E-cadherin***	***p=0.049***	***p=0.006***	***p=0.013***	***p=0.043***
	***r=0.167***	***r=-0.23***	***r=0.222***	***r=0.32***
***APC***	***p=0.614***	***p=0.231***	***p=0.251***	***p=0.68***
	***r=-0.051***	***r=-0.056***	***r=0.046***	***r=0.065***
***P53***	***p=0.203***	***p=0.003***	***p=0.756***	***p=0.438***
	***r=0.118***	***r=0.254***	***r=-0.229***	***r=0.121***
***COX-2***	***p=0.021***	***p=0.359***	***p=0.004***	***p=0.425***
	***r=0.206***	***r=-0.083***	***r=0.281***	***r=0.125***

Correlations of RIP140 and LCoR expression with clinical parameters in CRC and GC were summarized in Tables 4 and 5, respectively. In CRC, the only significant association with RIP140 expression was observed with TNM stage (p=0.027), whereas LCoR correlated significantly with age at diagnosis (p=0.019), tumor site (p=0.037) and differentiation (p=0.011). In GC, there were significant associations between RIP140 expression and TNM (p=0.001), tumor site (p=0.033), differentiation (p=0.005) and histotype (p=0.02), as shown in Table 5. Indeed, all diffuse tumors showed high RIP140 expression as compared to tumors of the intestinal type which displayed negative to moderate expression of RIP140 in 34.8% of the cases. Similarly, 100% of poorly differentiated tumors displayed high RIP140 expression compared to 60% of moderate to well differentiated tumors. Regarding LCoR expression, the only significant association was observed with TNM stage (p=0.042).

**Table 4:** Association between RIP140 and LCoR and clinicopathological features in colorectal cancer

*Parameters*	*n (102)*	*RIP140*			*n (99)*	*LCoR*		
		*Low n (%)*	*Moderate n (%)*	*High n (%)*		*Low n (%)*	*Moderate n (%)*	*High n (%)*
***Gender***								
***Male***	***47***	***6(12.8)***	***16(34)***	***25(53.2)***	***45***	***25(55.6)***	***8(17.8)***	***12(26.7)***
***Female***	***55***	***8(14.5)***	***14(25.5)***	***33(60)***	***54***	***29(53.7)***	***8(14.8)***	***17(31.5)***
***p****-****value***		***0.637***				***0.842***		
***Age***								
***≤60***	***39***	***3(7.7)***	***15(38.5)***	***21(53.8)***	***38***	***19(50)***	***11(28.9)***	***8(21.1)***
***>60***	***63***	***11(17.5)***	***15(23.8)***	***37(58.7)***	***61***	***35(57.4)***	***5(8.2)***	***21(34.4)***
***p****-****value***		***0.171***				***0.019***		
***TNM-stage***								
***I-II***	***41***	***2(4.9)***	***16(39)***	***23(56.1)***	***40***	***23(57.5)***	***5(12.5)***	***12(30)***
***III***	***42***	***6(14.3)***	***10(23.8)***	***26(61.9)***	***42***	***20(47.6)***	***7(16.7)***	***15(35.7)***
***IV***	***17***	***6(35.3)***	***3(17.6)***	***8(47.1)***	***17***	***11(64.7)***	***4(23.5)***	***2(11.8)***
***p****-****value***		***0.027***				***0.4***		
***Tumor site***								
***Colon***	***65***	***9(14.1)***	***16(25)***	***39(60)***	***64***	***38(59.4)***	***6(9.4)***	***20(31.3)***
***Rectum***	***37***	***5(13.5)***	***13(35.1)***	***19(51.4)***	***34***	***15(44.1)***	***10(29.4)***	***9(26.5)***
***p****-****value***		***0.544***				***0.037***		
***Differentiation***								
***Poor***	***61***	***6(9.8)***	***20(32.8)***	***35(57.4)***	***60***	***37(61.7)***	***8(13.3)***	***15(25)***
***Moderate***	***37***	***7(18.9)***	***8(21.6)***	***23(59.5)***	***37***	***17(45.9)***	***6(16.2)***	***14(36.8)***
***Well***	***2***	***1(50)***	***1(50)***	***0(0)***	***2***	***0(0)***	***2(100)***	***0(0)***
***p****-****value***		***0.22***				***0.011***		
***Tumor size***								
***T≤5 cm***	***53***	***11(16.4)***	***20(29.9)***	***36(53.7)***	***50***	***35(54.7)***	***10(15.6)***	***19(29.7)***
***T>5 cm***	***48***	***3(8.8)***	***10(29.4)***	***21(61.8)***	***48***	***19(55.9)***	***6(17.6)***	***9(26.5)***
***p****-****value***		***0.549***				***0.936***		

**Table 5:** Association between RIP140, and LCoR and clinicopathological features in gastric cancer

*Parameters*	*n (41)*	*RIP140*			*n (41)*	*LCoR*		
		*Low n (%)*	*Moderate n (%)*	*High n (%)*		*Low n (%)*	*Moderate n (%)*	*High n (%)*
***Gender***								
***Male***	***22***	***3(13.6)***	***2(9.1)***	***17(77.3)***	***22***	***9(40.9)***	***4(18.2)***	***9(40.9)***
***Female***	***19***	***1 (5.3)***	***2(10.5)***	***16(84.2)***	***19***	***6(31.6)***	***4(21.1)***	***9(47.4)***
***p****-****value***		***0.665***				***0.829***		
***Age***								
***≤60***	***19***	***3(15.8)***	***1(5.3)***	***15(78.9)***	***19***	***7(36.8)***	***4(21.1)***	***8(42.1)***
***>60***	***22***	***1(4.5)***	***3(13.6)***	***18(81.8)***	***22***	***8(36.4)***	***4(18.2)***	***10(45.5)***
***p****-****value***		***0.356***				***0.966***		
***TNM-stage***								
***I-II***	***2***	***0(0)***	***2(100)***	***0(0)***	***2***	***2(100)***	***0(0)***	***0(0)***
***III***	***10***	***1(10)***	***0(0)***	***9(90)***	***10***	***5(50)***	***3(30)***	***2(20)***
***IV***	***16***	***2(12.5)***	***1(6.3)***	***13(81.3)***	***16***	***2(12.5)***	***4(25)***	***10(62.5)***
***p****-****value***		***0.001***				***0.042***		
***Tumor site***								
***Antrum***	***23***	***7(30.4)***	***4(17.4)***	***12(52.2)***	***22***	***9(40.9)***	***4(18.2)***	***9(40.9)***
***Body***	***12***	***0(0)***	***6(50)***	***6(50)***	***12***	***1(8.3)***	***3(25)***	***8(66.7)***
***Cardia***	***4***	***1(25)***	***3(75)***	***0(0)***	***4***	***3(75)***	***1(25)***	***0(0)***
***p****-****value***		***0.033***				***0.094***		
***Differentiation***								
***Poor***	***21***	***0(0)***	***0(0)***	***21(100)***	***21***	***6(28.6)***	***3(14.3)***	***12(57.1)***
***Moderate-Well***	***20***	***4(20)***	***4(20)***	***12(60)***	***20***	***9(45)***	***5(25)***	***6(30)***
***p****-****value***		***0.005***				***0.215***		
***Histotype***								
***Intestinal***	***23***	***4(17.4)***	***4(17.4)***	***15(62.5)***	***23***	***11(47.8)***	***5(21.7)***	***7(30.4)***
***Diffuse***	***18***	***0(0)***	***0(0)***	***18(100)***	***18***	***4(22.2)***	***3(16.7)***	***11(61.1)***
***P****-****value***		***0.02***				***0.128***		
***HP***								
***Negatif***	***13***	***3(23.1)***	***4(30.8)***	***6(46.2)***	***13***	***7(53.8)***	***3(23.1)***	***3(23.1)***
***Positif***	***10***	***2(20)***	***3(30)***	***5(50)***	***10***	***1(10)***	***3(30)***	***6(60)***
***p****-****value***		***0.979***				***0.074***		
***Tumor size***								
***T≤5 cm***	***3***	***0(0)***	***0(0)***	***3(100)***	***3***	***0(0)***	***1(33.3)***	***2(66.7)***
***T>5 cm***	***31***	***3(9.7)***	***2(6.5)***	***26(83.9)***	***31***	***12(38.7)***	***6(19.4)***	***13(41.9)***
***p****-****value***		***0.753***				***0.407***		

### Association of RIP140 and LCoR expression with patient survival

**Figure 4:**
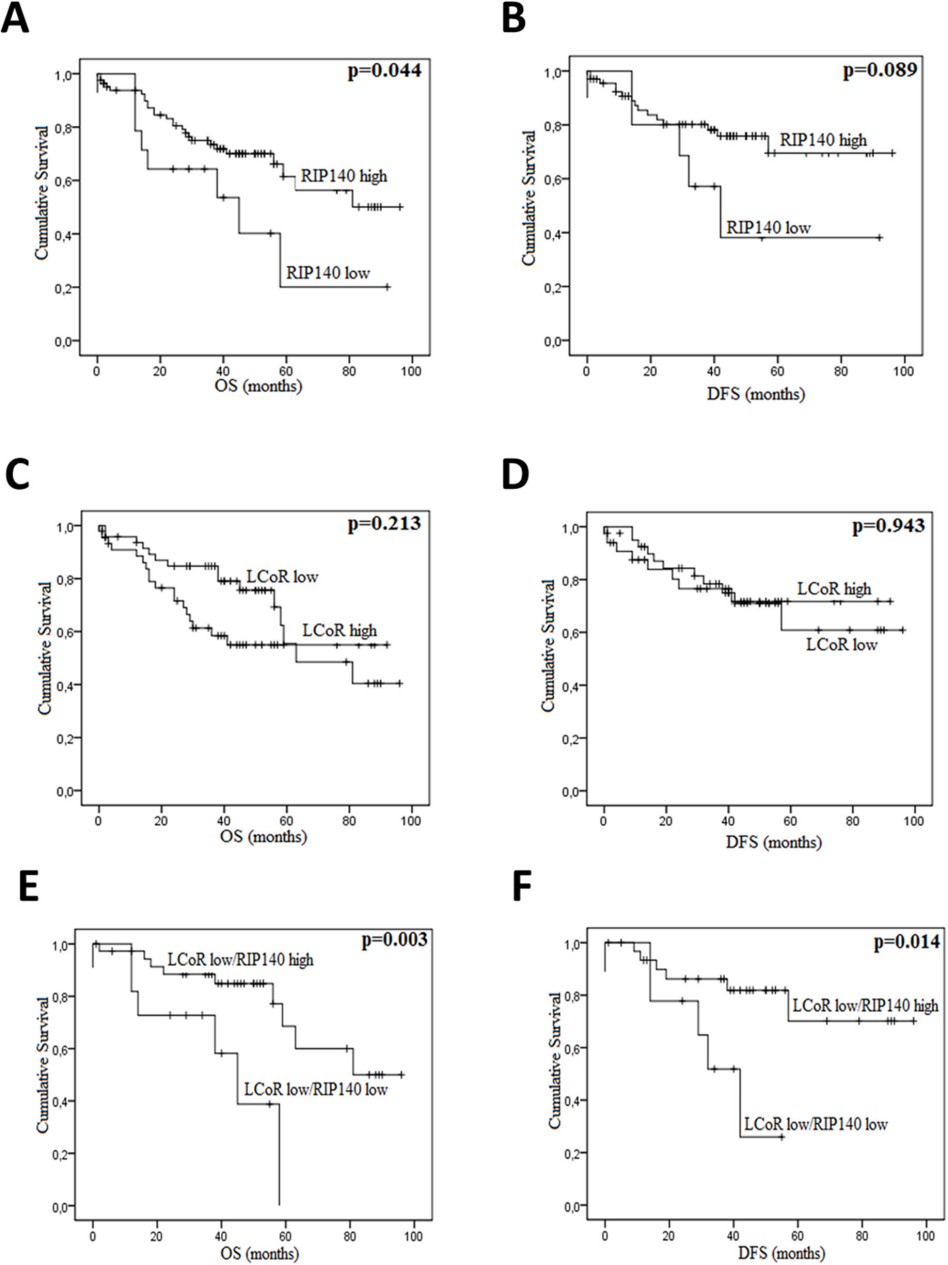
RIP140 and LCoR as prognosis markers in colorectal cancers Kaplan-Meier curves based on the levels of RIP140 **(A-B)** and LCoR **(C-D)** were drawn for OS **(A** and **C)** and DFS **(B** and **D)** on a cohort of 102 CRC patients. The same curves were drawn for OS **(E)** and DFS **(F)** taking into account both RIP140 and LCoR expression. The *p* values were calculated using the log-rank test and are indicated.

**Figure 5:**
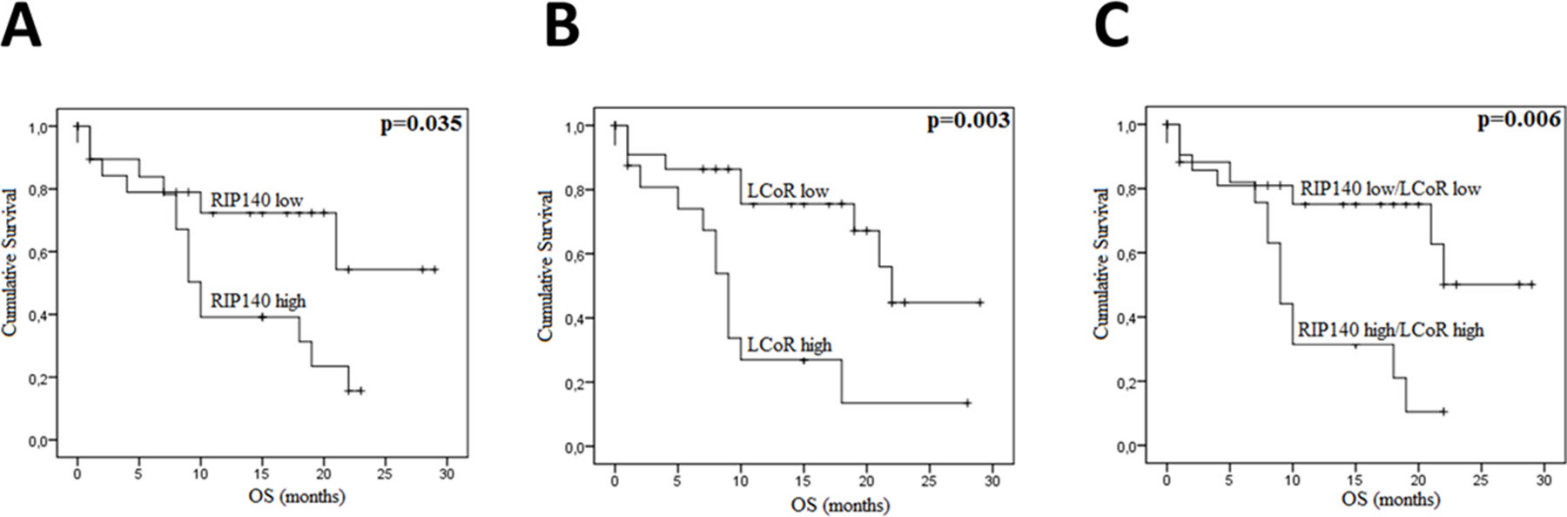
RIP140 and LCoR as a prognosis marker in gastric cancers Kaplan-Meier curves for overall survival (OS) in GC patients according to RIP140 **(A)**, LCoR **(B)** or both RIP140 and LCoR **(C)** expression in a cohort of 41 GC patients. The *p* values were calculated using the log-rank test and are indicated.

In CRC, the overall survival (OS) and disease-free survival (DFS) data were available for 99 and 80 patients respectively. Among 102 patients, 37 (35.2%) died from their disease. The median OS and DFS were 39.88 and 39.38 months, respectively. Kaplan-Meier survival curves were generated based on RIP140 and LCoR expression (Figure 4). The OS and DFS were longer for patients with tumors displaying high levels of RIP140 (Figure 4A and 4B), nevertheless a statistical significance was reached only for OS (p=0.044, Figure 4A). On the contrary, LCoR appeared likely to be not related neither to OS nor DFS in CRC (p=0.213, p=0.943 respectively) (Figure 4C and 4D). Interestingly, in the group of patients showing low LCoR immunostaining, the correlations of RIP140 expression with OS and DFS were stronger than in the whole population (p=0.003 and p=0.014, respectively) (Figure 4E and 4F). In GC, RIP140 as well as LCoR expression were significantly associated to OS but in an opposite manner to what occurred in CRC. Indeed, low expression of RIP140 (IS 0-2) conferred a benefit in terms of OS (p=0.035; Figure 5A). Likewise, the OS rate was longer for patients exhibiting low expression of LCoR compared to those with tumors showing high LCoR immunostaining (p= 0.003, Figure 5B). Interestingly, the significance of the correlation with OS was higher when patients with RIP140^low^/LCoR^low^ tumors were compared to the patients with RIP140^high^/LCoR^high^ tumors (p= 0.006, Figure 5C).

### Univariate and multivariate analyses of outcome predictors

Next, we conducted univariate and multivariate analyses using a Cox proportional hazard model to examine associations between age, sex, tumor location, differentiation, TNM stage, lymphovascular invasion (LVI), tumor size, RIP140 or LCoR expression and OS in CRC (Table 6). In univariate analysis, TNM stage predicted shorter OS (p = 0.001; 95% CI= 1.44- 3.76). Furthermore, differentiation and LVI were also associated with OS (p = 0.011, 95% CI, 1.23-4.86, p=0.007; 95% CI=1.30-5.10, respectively). In multivariate analysis, TNM stage (p=0.002; 95% CI=1.38-4.30), LVI (p=0.043, 95% CI=1.03-5.30), tumor size (p=0.012, 95% CI=1.25-6.3) and RIP140 expression (p=0.034, 95% CI=0.28-0.95) were independently related to OS after adjusting for age, sex, and LCoR expression (6).

**Table 6:** Univariate and multivariate Cox proportional hazard analysis of overall survival in CRC

*Variables*	*n*	*OS*			
		*Univariate*		*Multivariate*	
		*HR (95%CI)*	*p*	*HR (95%CI)*	*p*
***Age***			***0.208***		***0.064***
***<60***	***39***	***1.00***		***1.00***	
***≥60***	***63***	***1.58 (0.77-3.26)***		***2.1 (0.96-4.61)***	
***Sex***			***0.796***		***0.753***
***Male***	***47***	***1.00***		***1.00***	
***Female***	***55***	***1.09 (0.55-2.16)***		***1.13 (0.53-2.42)***	
***Tumor site***			***0.352***		***0.067***
***Colon***	***65***	***1.00***		***1.00***	
***Rectum***	***37***	***1.39 (0.69-2.78)***		***2.12 (0.95-4.71)***	
***Differentiation***			***0.011***		***0.638***
***Moderate-Well***	***39***	***1.00***		***1.00***	
***Poor***	***61***	***2.44 (1.23-4.86)***		***1.25 (0.49-3.24)***	
***TNM***			***0.001***		***0.002***
***I-II***	***41***	***1.00***		***1.00***	
***III-IV***	***59***	***2.33(1.44-3.76)***		***2.44 (1.38-4.30)***	
***LVI***			***0.007***		***0.043***
***No***	***67***	***1.00***		***1.00***	
***Yes***	***32***	***2.57 (1.30-5.10)***		***2.33 (1.03-5.30)***	
***Tumor size***			***0.327***		***0.012***
***<5cm***	***53***	***1.00***		***1.00***	
***≥ 5cm***	***48***	***1.42 (0.71-2.84)***		***2.81 (1.25-6.3)***	
***RIP140***			***0.073***		***0.034***
***Low***	***44***	***1.00***		***1.00***	
***Mod-High***	***58***	***0.58 (0.32-1.05)***		***0.51 (0.28-0.95)***	
***LCoR***			***0.416***		***0.103***
***Low***	***70***	***1.00***		***1.00***	
***Mod-High***	***29***	***1.17 (0.8-1.72)***		***1.41 (0.93-2.12)***	

Abbreviations: OS, overall survival; HR, hazard ratio; CI, confidence interval; CRC, colorectal cancer; LVI, lympho vascular invasion.

Although, the number of patients was small in GC (41 samples), univariate and multivariate analyses using a Cox proportional hazards model showed that some clinical parameters were related to patient survival (Table 7). Univariate analysis showed that age at diagnosis (p=0.025, 95% CI= 1.15-7.86), TNM stage (p=0.046, 95% CI= 1.02-6.95), expression ofRIP140 (p=0.039, 95% CI= 1.05-7.16) and LCoR (p=0.006, 95% CI= 1.47-10.02) correlated significantly with OS, whereas only LCoR associated with OS in multivariate analysis (p=0.004, 95% CI=1.67-14.6).

**Table 7:** Univariate and multivariate Cox proportional hazard analysis of overall survival in GC

*Variables*	*n*	*OS*			
		*Univariate*		*Multivariate*	
		*HR (95%CI)*	*p*	*HR (95%CI)*	*p*
***Age***			***0.025***		***0.227***
***<60***	***19***	***1.00***		***1.00***	
***≥60***	***22***	***3.01 (1.15-7.86)***		***1.93 (0.66-5.63)***	
***Sex***			***0.796***		***0.526***
***Male***	***22***	***1.00***		***1.00***	
***Female***	***19***	***1.12 (0.47-2.71)***		***0.71 (0.25-2.02)***	
***Histotype***			***0.766***		***0.834***
***Intestinal***	***23***	***1.00***		***1.00***	
***Diffus***	***18***	***0.87 (0.36-2.13)***		***0.84 (0.16-4.37)***	
***Differentiation***			***0.592***	***1.00***	***0.604***
***Moderate-Well***	***20***	***1.00***		***1.00***	
***Poor***	***21***	***1.27 (0.52-3.10)***		***0.75 (0.25-2.21)***	
***TNM***			***0.046***		***0.941***
***I-II***	***2***	***1.00***		***1.00***	
***III-IV***	***26***	***2.65 (1.02-6.95)***		***0.94 (0.20-4.36)***	
***RIP140***			***0.039***		***0.679***
***Low***	***22***	***1.00***		***1.00***	
***Mod-High***	***19***	***2.74 (1.05-7.16)***		***0.60 (0.05-6.77)***	
***LCoR***			***0.006***		***0.004***
***Low***	***25***	***1.00***		***1.00***	
***Mod-High***	***16***	***3.84 (1.47-10.02)***		***4.94 (1.67-14.6)***	

Abbreviations: OS, overall survival; HR, hazard ratio; CI, confidence interval; GC, gastric cancer. A backward elimination with threshold of *P*=0.6 was to select variables in the final models.

## DISCUSSION

The role of RIP140 in colon tumor progression and its cross-talk with the Wnt/β-catenin signaling has been previously reported, together with its decreased expression in CRC and its correlation with good prognosis [25]. In the present study, we further decipher by IHC, the expression of RIP140 in colon carcinomas and analyzed, for the first time, the expression of the *LCOR* gene (recently identified as a RIP140 interactor and target gene) in this malignancy. This work is also the first study which analyzed the relevance of these two transcription factors in gastric cancer.

Our data first demonstrate a significant positive association between *RIP140* and *LCOR* gene expression both in colon and gastric cancers (Figure 2). This is in perfect line with a recent study showing that the expression of the two genes were highly correlated in breast cancer cells and tumors [26]. Recently, the same observation has been made in cervical cancers (U Jeschke, unpublished observations). It thus appears that *RIP140* and *LCOR* gene expression is strongly correlated in different cancer types. In breast cancer cells, it was demonstrated that RIP140 was able to transactivate the *LCOR* gene promoter [26]. In GICs, the exact mechanisms which account for the correlation remain unknown but preliminary experiments using luciferase reporter assays in colon and gastric cancer cell lines indicate that RIP140 exerts a positive effect on *LCOR* gene transcription (S. Jalaguier and M. Triki, unpublished observations).

The present study also showed that both *RIP140* and *LCoR* gene expression is deregulated in GICs (Figure 3). The IHC staining for both RIP140 and LCoR decreased in colorectal tumors as compared to adjacent normal tissue which is, for RIP140, in agreement with a previous study [25]. On the contrary, in GC significant higher levels of RIP140 and LCoR proteins were detected in tumors compared to adjacent normal tissues. RIP140 overexpression in GC was confirmed by reanalyzing the GDS1210 transcriptomic data available on the GEO profile database [33] (see Supplementary Figure 2). In breast cancers, RIP140 and LCoR mRNA expression were also found significantly higher in breast tumor samples as compared to normal mammary glands [26]. The difference in the patterns of *RIP140* and *LCOR* gene expression in CRC compared to GC may reflect the complexity by which the two genes are regulated both at the transcriptional and post-translational levels. Several studies have reported data concerning the regulation of RIP140 expression and post-translational modifications [34] [35] whereas little is known concerning *LCOR* gene regulation in cancer cells and tissues.

It should be noted that RIP140 is engaged in several transcriptional regulatory feed-back loops involving nuclear receptors or E2F1. The regulation of RIP140 (and possibly LCoR) gene expression by these transcription factors could be at the basis of their differential expression in GC *vs* CRC. Moreover, the regulation by other signaling pathways (including Wnt, Notch, Hippo and others) might also participate in these normal vs cancer dysregulations.

Regarding the prognosis relevance of RIP140 and LCoR in CRC and GC, Kaplan Meier plots showed that high expression of RIP140 conferred a benefit in terms of OS and DFS in CRC patients (Figure 4A and 4B), which is concordant with a previous study [25]. Although the expression of the two genes was strongly correlated, it appeared that LCoR expression was rather associated with poor prognosis in CRC. In line with this observation, we noticed that the association of RIP140 expression with both OS and DFS of CRC patients was stronger in tumors expressing low levels of LCoR (Figure 4E and 4F). It has been reported that the two transcription factors could interact and act synergistically [26]. The present data may indicate that, in CRC cells, LCoR could rather antagonize RIP140 activity.

Some of the molecular mechanisms controlling tumorigenesis and involving RIP140 have been elucidated for breast and colon cancers. In breast cancer, both *in vitro* and *in vivo* studies indicate that RIP140 exerts direct transcriptional control on signaling pathways including nuclear receptors and E2F transcription factors (for a review see [36]). In colon cancer, RIP140 interferes with the Wnt/β-catenin pathway through positive regulation of the tumor suppressor gene APC which in turn drives the degradation of β-catenin [25]. It should be noticed that no correlations between RIP140 and APC expression were observed in the CRC cohort used in this study (See Table 3) suggesting that the relationship between RIP140 and the Wnt/β-catenin might be complex and that further investigations are needed to better elucidate this connection.

The difference in the correlation with survival of RIP140 and LCoR in CRC compared to GC may reflect the different contribution of the two transcriptional coregulators to tumor development and progression in these epithelia. Very interestingly, RIP140 associated positively with E-cadherin in CRC whereas LCoR exhibited a significant negative correlation (Table 3). E-cadherin is a transmembrane glycoprotein which plays an important role in maintaining the structural integrity of epithelial sheets [37]. The loss of E-cadherin expression has been reported in several GIC including advanced colorectal carcinomas [29] [30] [37].

Interestingly, experiments used to silence the expression of E-cadherin not only showed a morphological shift from an epithelial to a fibroblastoid phenotype, characteristic of EMT, but also a concomitant increase in invasive cell behavior [38]. The loss of E-cadherin has been considered to increase cellular dissemination and tumor metastasis. The different correlation of RIP140 and LCoR with E-cadherin expression may therefore suggest different roles on CRC metastasis and represent a clue to explain their correlation with good and bad prognosis, respectively.

**Figure 6:**
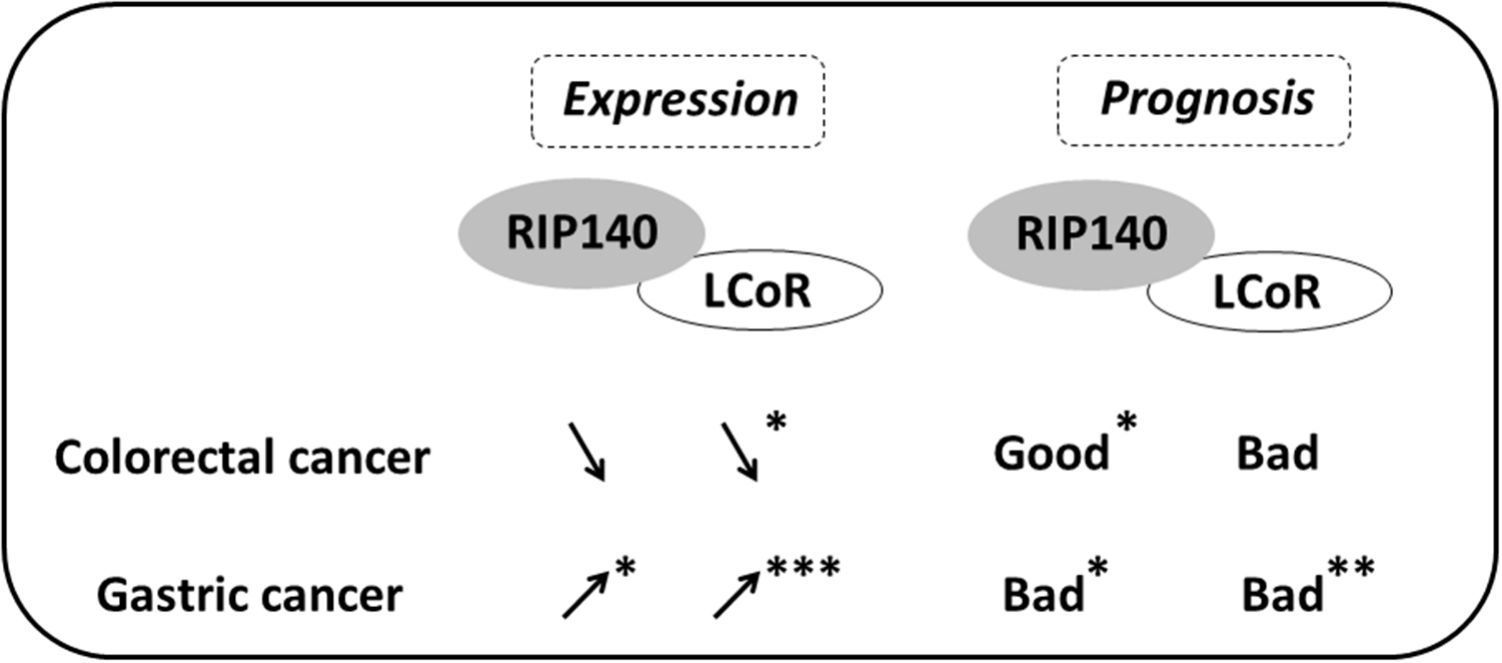
Scheme comparing the expression and prognosis value of RIP140 and LCoR in CRC and GC The variations of RIP140 and LCoR expression in tumoral tissue as compared to normal mucosa are shown by arrows for CRC and GC. The prognostic value (bad or good) of RIP140 and LCoR expression is also shown. Significant correlations are marked with asterisks (^*^=p≤0.05, ^**^=p≤0.01, ^***^=p≤0.001).

Concerning GC, the ways RIP140 and LCoR are involved in the development and progression of this malignancy remain to elucidate. It could be hypothesized that other signaling pathways besides Wnt/β-catenin could be involved and might include, for instance, the Hippo pathway [39]. Interestingly, our data indicated a positive association between RIP140 and COX-2 both in CRC and GC (Table 3) suggesting a relationship between these two proteins involved in inflammation, as previously reported [40]. In GC, high expression of RIP140 (alone or in combination with LCoR *ie* RIP140^high^/LCoR^high^ tumors) correlated with shorter OS rate in strong opposition with what was observed in CRC. To emphasize these results, we reanalyzed public datasets of GC using the Kaplan Meier plotter (http://kmplot.com) which allows meta-analysis based biomarker assessment using a background database which is manually curated [41]. This confirmed that both RIP140 and LCoR expression were significantly correlated with poor prognosis in GC patients (see Supplementary Figure 3). This difference in survival prognosis in CRC and GC is in perfect accordance with the opposite dysregulation of their expression between normal and tumoral epithelium in the two malignancies (see Figure 3 and Figure 6). Finally, the most important observation is that RIP140 and LCoR appeared as independent predictors of patient survival in multivariate analyses, RIP140 being a marker of good prognosis in CRC whereas LCoR is a marker of poor prognosis in GC (Tables 6 and 7). In conclusion, our data demonstrate that the expression of RIP140 and LCoR have a prognostic relevance in GICs and could represent new potential biomarkers or targets in these tumors.

## MATERIALS AND METHODS

### Patients and tumor samples

We obtained a total of 189 tissue specimens from 143 patients including 125 colorectal tissues (102 cancers and 23 matched normal surgical margins) and 64 stomach tissues (41 cancers and 23 matched normal surgical margins). These samples were collected at the Department of Digestive Surgery of the Habib Bourguiba University Hospital (Sfax, Tunisia) from 1995 to 2012. All patients gave informed consent prior to specimen collection according to institutional guidelines. None of the patients had pre-operative or post-operative chemotherapy. Patient information were obtained from medical records and included age, gender, differentiation grade, tumor site, size and stage, histological type and *H. pylori* infection (Table 1). The histological subtypes were classified according to criteria of the World Health Organization. The tumors were staged according to the TNM (tumor, lymph node and metastases) classification adopted by the American Joint Committee on Cancer [42]. The median follow-up period was 38 months (0-96 months) for CRC patients and 9 months (0-29 months) for GC patients. Follow-up data on survival rate was available for all cases of GC and only in 96 cases of CRC.

### Immunohistochemical staining

The samples obtained at surgery were routinely fixed in 10% neutral buffered formalin and embedded in paraffin. Before immunostaining, two pathologists (LA, SC) reviewed haematoxylin and eosin-stained slides in order to select blocks representing invasive adenocarcinoma. For each selected tumor, 4-μm sections attached on poly-L-lysine-coated slides were fixed in acetone for 10 min, and left to dry overnight at 37 °C. Slides were deparaffinized in xylene followed by subsequent rehydration in graded ethanol. The sections were then pre-treated with 3% hydrogen peroxide for 10 min to inactivate endogenous peroxides and washed in phosphate-buffered saline (PBS) solution. Heat-induced antigen retrieval was performed using epitope retrieval solution (Dako) at 95 °C for 40 min. After heating, slides were allowed to cool down to room temperature and were briefly washed with PBS. Blocking solution (Dako) was used for 5 min to block the non-specific binding of antibodies. Immunohistochemical staining was performed using the streptavidin–biotin peroxidase system (RE7280-K, Leica Biosystems). Tissue sections were incubated overnight with the primary antibody against RIP140 (ab42124, Abcam) or for 30 min with the anti- LCoR antibody (ab48339, Abcam). Primary antibody binding was visualized with biotin- labeled secondary antibodies (Novolink Polymer, Leica Biosystems) and a streptavidin– peroxidase complex using diaminobenzidine as a chromogenic substrate (RE7280-K, Leica Biosystems). Stainings with the other antibodies directed against APC (*adenomatous polyposis coli*), β-catenin, p53, COX-2 and E-cadherin were described elsewhere for the same CRC [28] [29] and GC biopsies [30] [31] [32].

### Immunostaining scoring

Immunostainings were scored on the basis of the percentage of positive tumor cells and the relative immunostaining intensity [43]. The initial scoring was graded according to the extent of immunostaining as follows: 0, no staining or less than 5% of tumor cells labelled; 1, 5-25% staining; 2, 25-75% staining; 3, 75-100% staining. The staining intensity was evaluated as follows: 0, negative; 1, low; 2, moderate; and 3, high. The final score was determined by multiplying the intensity of positivity and the extent of immunostaining yielding an immunoscore (IS) that ranged from 0 to 12. The immunostaining was determined as positive or negative by a cutoff value. RIP140 and LCoR were interpreted as low (IS: ≤2), moderate (IS>2 and ≤ 4) and high (IS> 4).

### Statistical analysis

Statistical analyses were performed using the SPSS 20.0 statistical software for Windows (SPSS Inc., IBM). Chi2 test was used to correlate the IS with clinicopathological features. Survival was estimated using the Kaplan-Meier method and log-rank test. Cox proportional hazard regression models were used to determine univariate and multivariate HRs. Statistical analysis and graphs were performed using the nonparametric Mann–Whitney *U* test with GraphPad Prism 7.0 (Ritme Informatique). Data are expressed as boxplots showing the median and interquartile range (box) and the 10–90 percentiles (whiskers). Statistical differences were assumed significant for p < 0.05 for both tests.

## SUPPLEMENTARY MATERIALS FIGURES



## Abbreviations

APC: Adenomatous polyposis coli
CI: confidence interval
CRC: colon cancer
DFS: disease free survival
EMT: epithelial mesenchymal transition
GC: gastric cancer
GIC: gastrointestinal cancer
IHC: immunohistochemistry
IS: immunoscore
KLF6: Kruppel-like factor 6
KAP1: KRAB-associated protein 1
LCoR: ligand dependent corepressor
LVI: lymphovascular invasion
OS: overall survival
PBS: phosphate-buffered saline
RIP140: receptor interacting protein of 140 kDa
TNM: tumor, lymph node and metastases
