# Supplementary material for: RIP140 and LCoR expression in gastrointestinal cancers
Source: Oncotarget. 2017 Nov 25;8(67):111161–75. doi: 10.18632/oncotarget.22686 (PMC5762313; doi:10.18632/oncotarget.22686)
Supplement: Supplementary file 1 [file oncotarget-08-111161-s001.pdf]

RIP140 and LCoR expression in gastrointestinal cancers

SUPPLEMENTARY MATERIALS

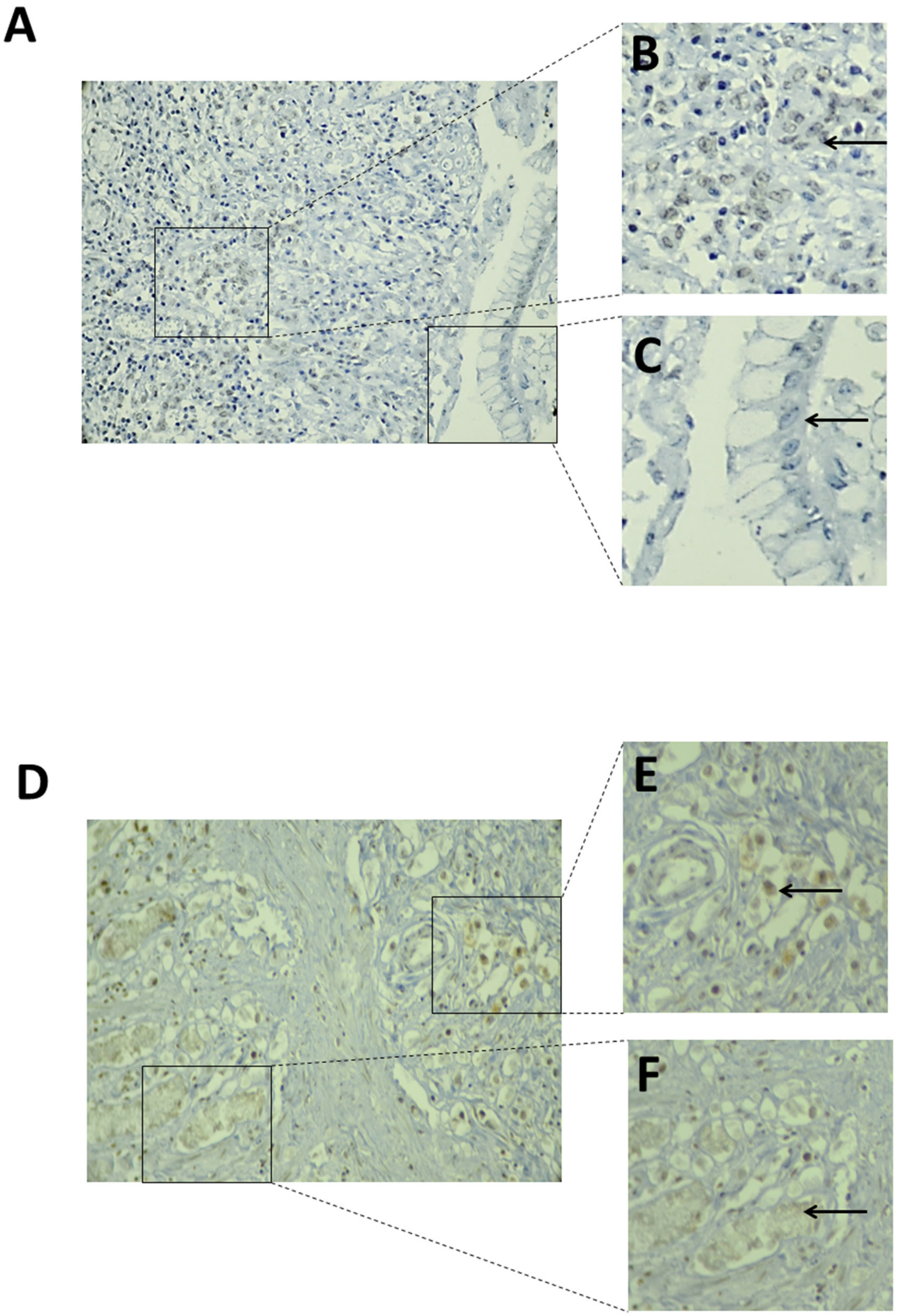

Supplementary Figure 1: Increased expression of RIP140 and LCoR in gastric cancer. Immunostaining of RIP140 **(A)** and LCoR **(D)** expression in tumoral gastric cancer **(B** and **E)** in comparison to normal mucosa **(C** and **F)**.

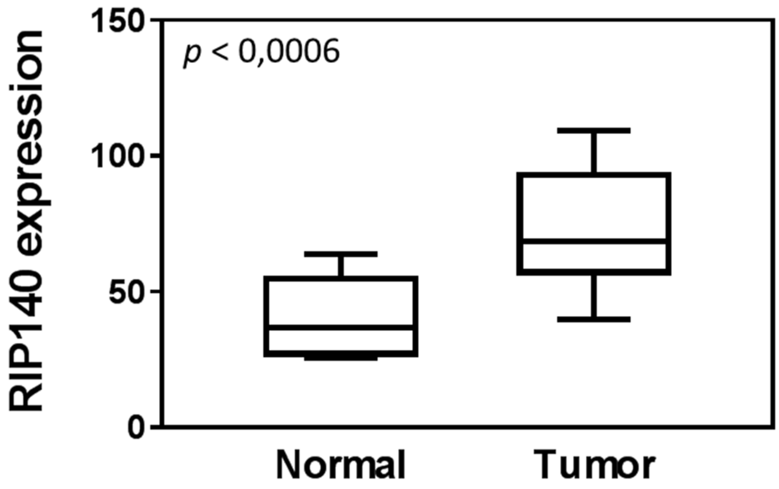

Supplementary Figure 2: Higher levels of RIP140 mRNA in GC. Reanalysis of the GDS1210 transcriptomic data showing the up-regulation of RIP140 expression in gastric cancer compared to normal mucosa. Correlations were performed using the nonparametric Mann–Whitney *U* test. Boxplots show the median and interquartile range (box) and the 10-90 percentiles (whiskers).

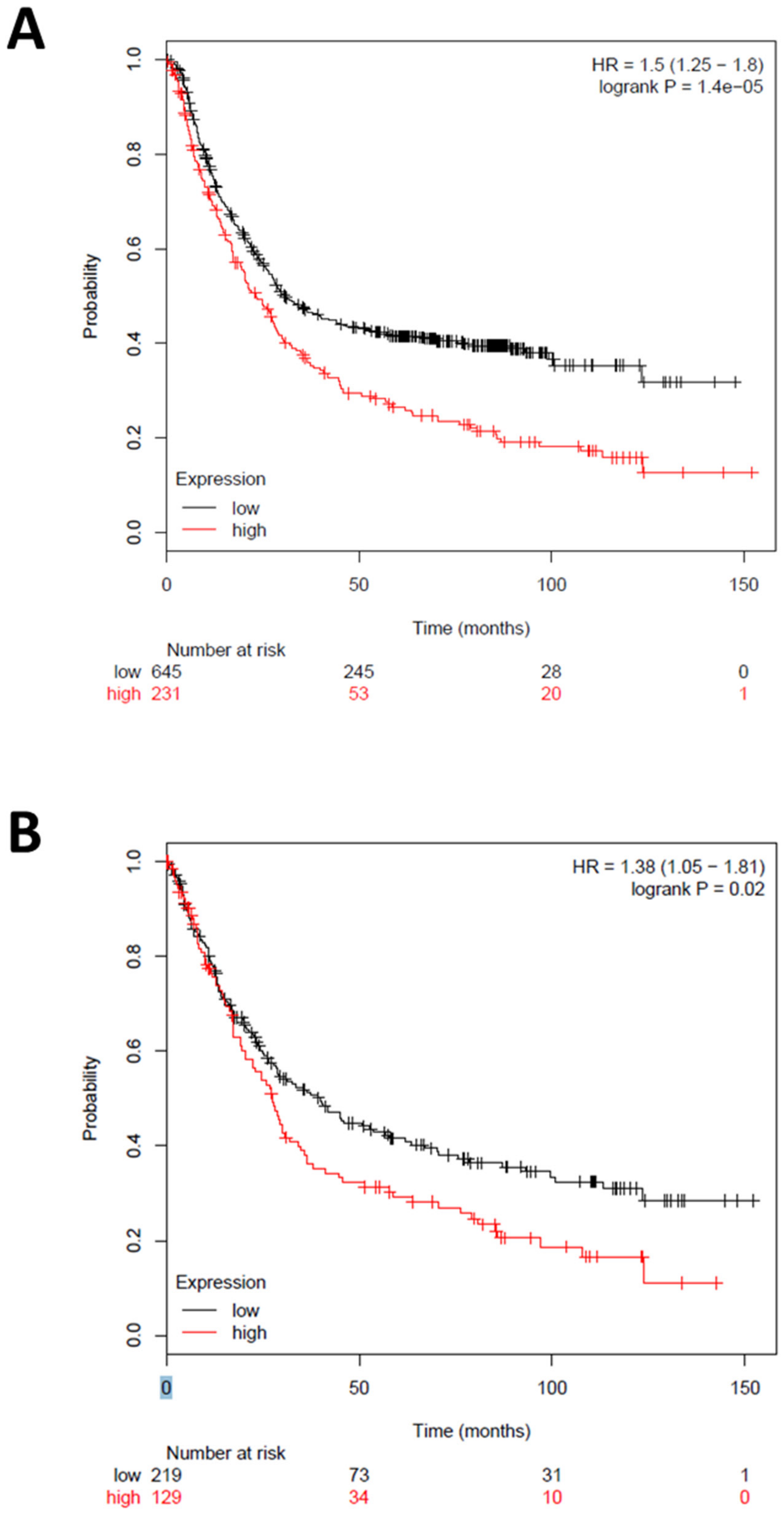

Supplementary Figure 3: Correlation of RIP140 and LCoR mRNA expression with OS of patients with GC. The Kaplan Meier plotter [41] was used to analyze the correlation of RIP140 and LCoR gene expression (using median cut-off) with overall survival of patients with GC. **(A)** Kaplan Meier curve for the RIP140/NRIP1 probeset 202600_s_at in 876 patients. **(B)** Kaplan Meier curve for the LCoR probeset 226520_at in 348 patients. The hazard ratio with 95% confidence intervals and log-rank p-value were calculated.
